# Electron-beam patterned calibration structures for structured illumination microscopy

**DOI:** 10.1038/s41598-022-24502-0

**Published:** 2022-11-23

**Authors:** Sangeetha Hari, Johan A. Slotman, Yoram Vos, Christian Floris, Wiggert A. van Cappellen, C. W. Hagen, Sjoerd Stallinga, Adriaan B. Houtsmuller, Jacob P. Hoogenboom

**Affiliations:** 1 Imaging Physics, Delft University of Technology, Delft, The Netherlands; 2 Department of Pathology, Erasmus Optical Imaging Centre, Erasmus Medical Center, Rotterdam, The Netherlands

**Keywords:** Super-resolution microscopy, Surface patterning

## Abstract

Super-resolution fluorescence microscopy can be achieved by image reconstruction after spatially patterned illumination or sequential photo-switching and read-out. Reconstruction algorithms and microscope performance are typically tested using simulated image data, due to a lack of strategies to pattern complex fluorescent patterns with nanoscale dimension control. Here, we report direct electron-beam patterning of fluorescence nanopatterns as calibration standards for super-resolution fluorescence. Patterned regions are identified with both electron microscopy and fluorescence labelling of choice, allowing precise correlation of predefined pattern dimensions, a posteriori obtained electron images, and reconstructed super-resolution images.

## Introduction

Structured illumination microscopy (SIM) achieves sub-diffraction limited resolution under illumination conditions compatible with live-cell imaging. In SIM, data is reconstructed from a stack of images recorded after modulation with mutually rotated and shifted inhomogeneous illumination patterns. Data reconstruction is computationally intensive and various SIM reconstruction algorithms have been reported over the past years^1–8^. These algorithms involve parameters, for instance for regularization and apodization filters, that are generally adjusted at will by the user. As a result, the reconstruction algorithms can give rise to artefacts that, owing to the structural complexity of biological samples, may be hard to differentiate from features of interest. Recognition and mitigation of artefacts has become subject of scientific debate^9,10^.

Performance testing for reconstruction algorithms for super-resolution microscopy is typically done using simulated data sets^11^. Results may, however, differ for data collected under experimental conditions, possibly on microscopes with sub-optimal alignment leading to optical aberrations^12^. Moreover, calibration and performance monitoring of SIM microscopes needs experimental standards, which should cover all relevant lengths scales, i.e. from the ~ 100 nm improved resolution limit to > 10 µm in lateral dimensions. To generate fluorescent patterns reliably over these dimensions, a position accuracy around 10 nm is needed. In addition, pattern dimensions need to be assessable by an independent technique with higher intrinsic resolution than SIM. Current schemes for generating commercial light microscopy calibration standards rely on one- or two-photon optical lithography^13^, which does not reach the desired sub-diffraction limited resolution. Point spread function evaluation can be done using individual precisely defined, highly monodisperse fluorescent nanobeads or refractive-index matched colloidal crystal structures^14^ with randomly dispersed fluorescent beads. Evaluation of the full, multi-scale modulation transfer function (MTF) however still poses challenges, e.g. due to the finite size of the nanobead or the requirement to record a full through-focus image stack. Similarly, molecular localization accuracy in techniques like PALM and STORM can be assessed using nanorulers that consist of single fluorophores with nanometer-range controlled separation using DNA-origami technology^15^, but the extension of DNA-based rulers to cover micrometer or larger length scales has remained elusive. Thus, the generation of pre-defined, complex fluorescent patterns that meet the requirements for calibration and algorithm testing for SIM microscopy remains a key challenge in super-resolution microscopy.

Here, we present super-resolution calibration samples using electron beam patterning in combination with targeted immuno-labelling of the electron-exposed areas. Multiple, predefined patterns are generated in a PEG monolayer on an ITO-coated glass substrate by exposure to a scanning electron beam. The exposed areas can then be made fluorescent upon incubation with a solution of immunoglobulin (IgG) antibody coupled to a fluorescent dye^16^. In their original work, Schlapak et al. attributed the functionalization to electron-beam induced carbon deposition on the exposed areas, mitigating the anti-fouling property of the PEG^16^. We adopted this procedure and developed an optimised scheme to reproducibly create patterns suitable for super-resolution microscopy calibration. We further show that patterned areas are visible in secondary electron (SE) contrast in the scanning electron microscope (SEM). By exploiting the adaptability of the IgG anti-body to other labels we created patterns of colloidal quantum dots, allowing independent evaluation of pattern dimensions, crucial for use in super-resolution microscopy.

## Results and discussion

### Pattern generation and validation

**Figure 1:**
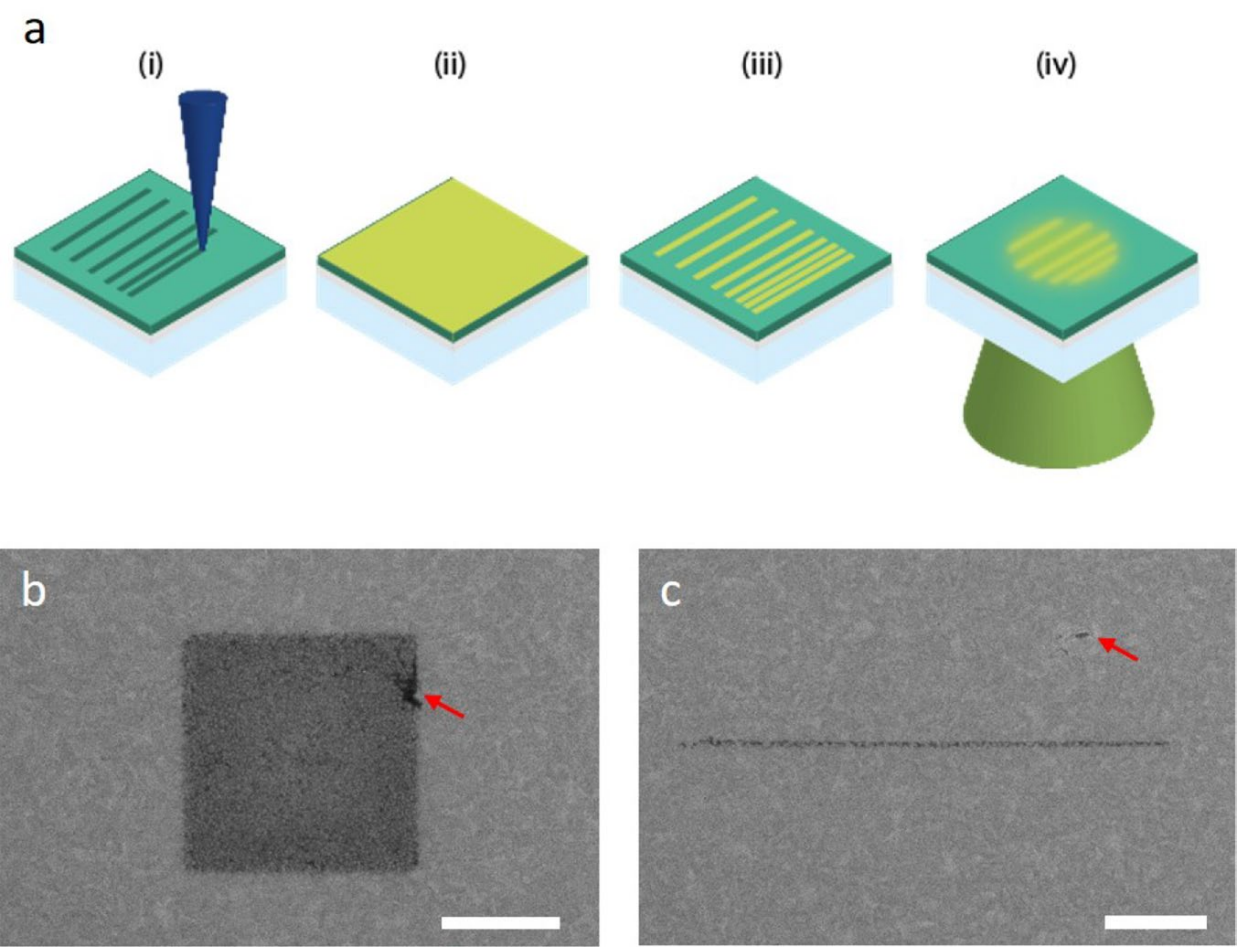
Electron-beam patterning and dimension control of fluorescent nanostructures for super-resolution calibration (**a**) Schematic illustration of the patterning procedure: (i) a scanning focused electron beam (depicted in blue) is used to create nanoscale patterns in a PEG monolayer on an ITO-coated glass slide, (ii) the patterned slides are incubated in a buffer solution containing IgG anti-body linked fluorescent molecules or quantum dot nanoparticles, (iii) slides are washed with buffer and deionised water to remove non-specifically bound antibodies before drying, and (iv) inspected with a fluorescence microscope. (**b**,**c**) In the electron microscope, after step (i), patterned areas are visible as dark contrasted regions allowing direct evaluation of the patterning procedure and the pattern dimensions. Variations in grayscale contrast in both square and line pattern are indicative of the stochastic variations in local electron dose. Red arrows indicate small defects (**b**) in the patterned area or (**c**) on the PEG layer that may compromise pattern or background fluorescence respectively. Scale bars are 1 µm.

The patterning procedure is briefly summarized in Fig. 1a, a detailed, step-by-step protocol is listed in the ‘Methods’ section. To obtain smooth PEG layers on ITO and high-resolution patterns with minimal defects we found the homogeneity of the ITO coating layer to be of critical importance (see also Supplemental Information). After grafting the PEG-silane monolayer to the ITO glass slide, the sample was mounted in the SEM and patterning was started as soon as the chamber had reached a pressure of 1 × 10^–5^ mbar. Care was taken to minimise vibrations (typically < 5 nm) and patterning was carried out with a 25 pA, 5 keV electron beam. Exposed areas, here with a fluence of 10 C/m^2^, are clearly visible by SE contrast (Fig. 1b,c), indicative of modification of the PEG surface or deposition of amorphous carbon. Both square and line patterns display speckle-like and grayscale variations in SE contrast, indicative of stochastic variations in the local electron dose. The exposed areas are fluorescently functionalized by incubation in a solution containing antibody-linked fluorescent molecules or nanoparticles.

**Figure 2:**
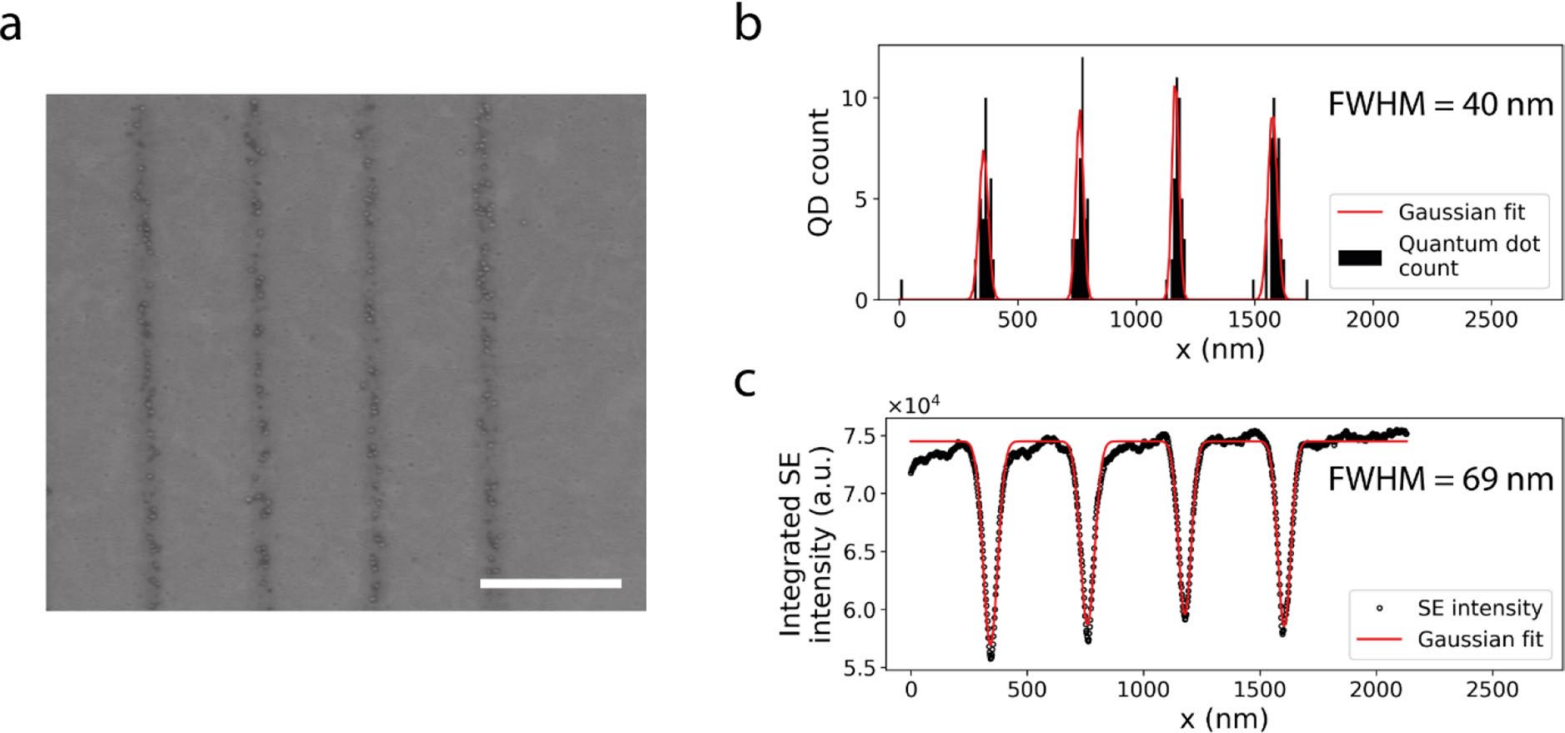
Quantum dot labelling confirms a posteriori obtained pattern dimensions. (**a**) Electron microscopy image of single quantum dot wide lines showing the quantum dot binding locations coincide with the darker contrast observed from the patterned areas in secondary electron mode. Distribution of (**b**) quantum dots positions and (**c**) secondary electron contrast obtained by integration along the direction of the lines. We obtain a line width of 40 nm for the quantum dots positions which falls within the 69 nm linewidth obtained on the secondary electron contrast. Scale bar in (**a**) is 500 nm.

Colloidal quantum dots were used to evaluate the dimensions of functionalized areas and their relation to the SE contrast. Quantum dots tagged with IgG were chosen because of the ease of fluorescence inspection for evaluating successful functionalization and the ability to visualize them with SEM for high-resolution dimensional analysis. Moreover, we obtained higher labelling densities with quantum dots than with fluorescently labelled gold nanoparticles, in agreement with previous reports^17^. Figure 2a clearly shows our ability to pattern single lines consisting of quantum dots. Individual quantum dots can be clearly discerned and their positions are seen to overlap with the appearance of darker SE contrast from the substrate. Moreover, regions along the lines where we observe no binding of quantum dots coincide with positions where the SE contrast along the line can hardly be distinguished from that of the unexposed neighbouring area. Thus, we conclude that no binding occurs outside of regions with SE contrast and that pattern dimensions can thus be evaluated directly after pattern writing in the SEM.

The functionalized line width was assessed by retrieving the positions of individual quantum dots and binning the number of quantum dots in the direction perpendicular to the lines. The resulting Gaussian line profile shows a full width at half maximum (FWHM) of 40 nm (Fig. 2b), well below the diffraction limit of visible light for wide field (WF) microscopy. The estimated FWHM corresponds well with a 69 nm line width evaluated for bare SE contrast on lines written under similar conditions but without functionalization (Fig. 2c), where the slight broadening might be due to the size of the antibody-quantum dot construct. As these dimensions are well matched to the expected resolution limit of SIM, which is about half the diffraction limit, we set out to create fluorescent patterns suitable for evaluation and calibration of SIM microscopes.

### Fluorescent nanopatterns for microscope calibration

**Figure 3:**
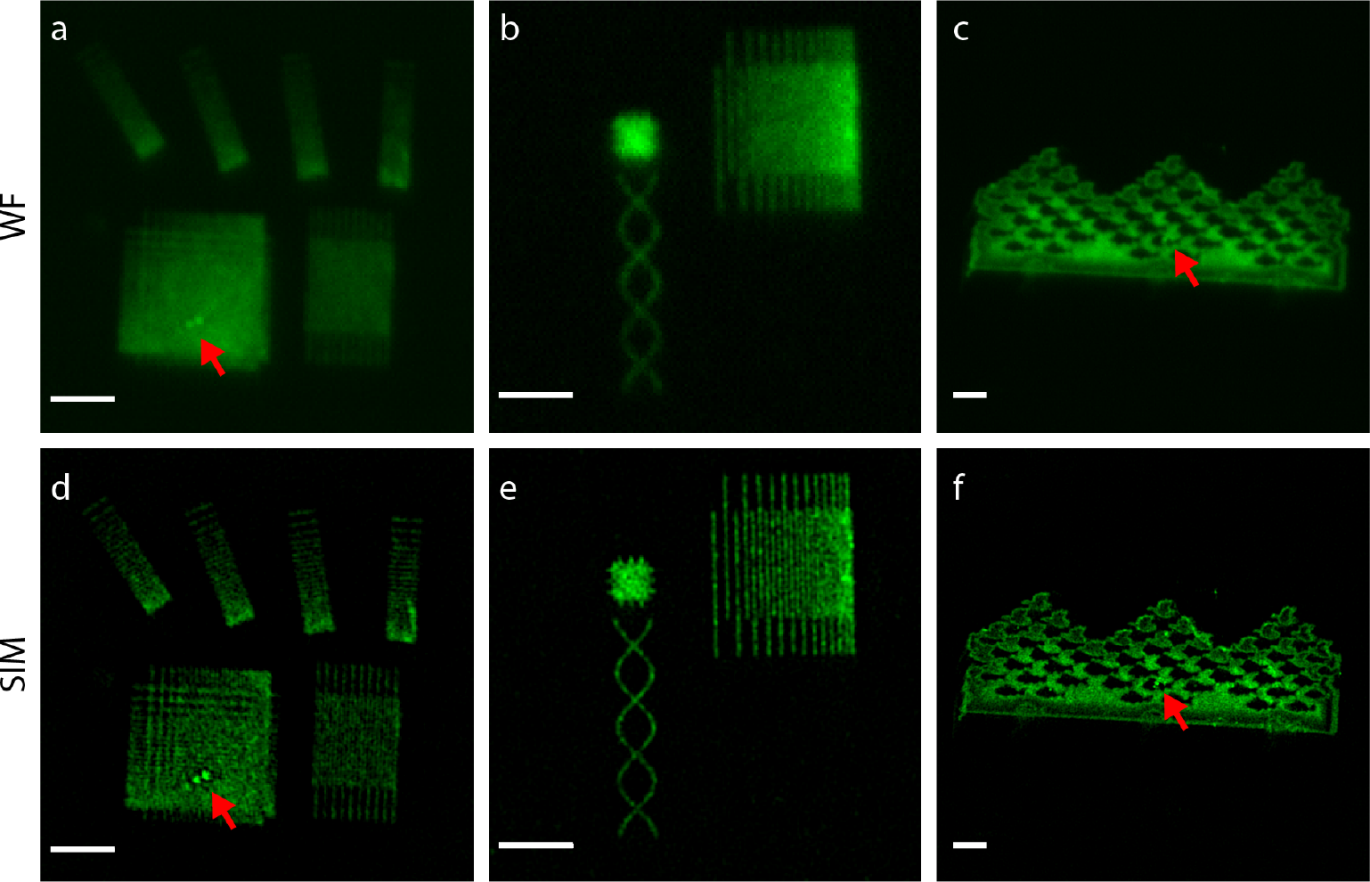
Fluorescent nanopatterns for super-resolution microscope calibration. (**a**–**c**) Wide field fluorescence microscopy images of the collection of patterns written for assessing microscope performance, including (**a**) lines with from top to bottom decreasing line spacing under different orientation, a checkerboard pattern with in two dimensions decreasing line spacing, interdigitated lines with equal spacing, (**b**) interdigitated lines with from left to right decreasing spacing, a solid square with protruding small lines, a double helix, and (**c**) a nanoscale version of M.C. Escher’s Sky and Water I. Pattern positions on the microscope slide were recognized by sequences of larger arrays of solid fluorescent squares (not shown). (**d**–**f**) Structured illumination microscopy images of the patterns in (**a**–**c**). The improvement in resolution is apparent from the various line patterns and the protruding lines in the small square, but also known reconstruction artefacts like enhanced noise appear for instance around the double helix structure. Red arrows point to small defects in the fluorescent patterns. Scale bars are 2 µm.

For SIM, we created patterns composed of lines with decreasing centre-to-centre spacing, line patterns under different rotation angle, and several other patterns of varying complexity, including a miniature version of “Sky and Water I” by M. C. Escher (Fig. 3). To illustrate adaptation to other labels and to match our SIM excitation and emission channels, quantum dots were replaced by Alexa488. All functionalized patterns are recognized by bright fluorescence. A few localized defects and aggregates giving rise to strong fluorescence can be recognized in some of the patterns. We also observe stochastic variations in fluorescence signal along the lines, which may be due to sparsity in labelling along the lines as observed above for the quantum dots. Wide-field microscopy is clearly limited in resolution, evidenced in visible blurring, lack of ability to distinguish closely spaced lines, and loss of fine details (Fig. 3a–c). The improved resolution for SIM is markedly visible for all patterns (Fig. 3d–f).

**Figure 4:**
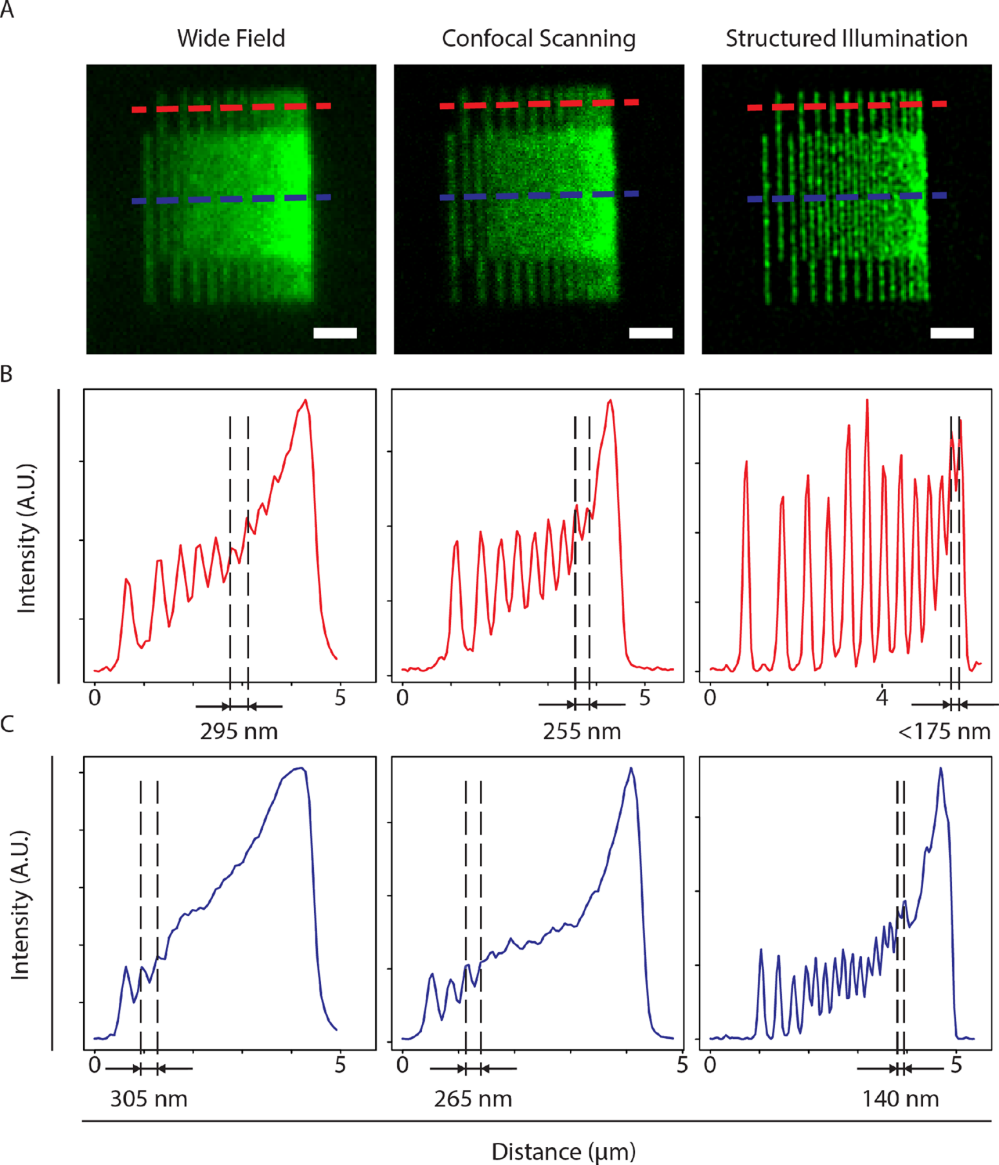
Fluorescent lines patterns confirm improved SIM resolution. (**A**) Wide field, confocal laser scanning, and structured illumination microscopy images of the interdigitated lines pattern with decreasing spacing. Intensity profiles recorded along the red and the blue dashed lines in (**B**) and (**C**) respectively. For all imaging modes, the intensity in between lines increases upon reaching the resolution limit. Dashed lines and numeric values indicate the smallest observed line spacing on all three images, in correspondence with the expected resolution improvement from WF to CLSM and SIM respectively. Sale bars in (**A**) are 1 µm.

We quantified the resolution improvements between WF microscopy, confocal laser scanning microscopy (CLSM), and SIM using the pattern with decreasing, interdigitated line spacing (Fig. 4). These images clearly show the improvements in resolution obtained with the latter two techniques. Individual lines can be clearly discerned with a smaller line width from WF to CLSM to SIM. For all three modalities, intensity between the lines increases on approaching the resolution limit. Quantitative assessment of line separation indicates resolution limits around 295 nm, 255 nm, and 140 nm for WF, CLSM, and SIM respectively.

Patterns imaged in WF or SIM mode can be imaged repeatedly. We stored samples at 4 °C, which ensured the signal remained intact between measurements. Thus, these patterns can serve as a calibration sample for microscope alignment, to monitor microscope performance in time for different fluorophores or to compare results and consistency between different microscopes. Moreover, thanks to the fact that patterns extend over a wide (100 nm–100 µm) size range and are defined with regular features and well-defined edges, they can be used for evaluation of the performance of SIM reconstruction algorithms.

### Assessing SIM reconstruction performance

**Figure 5:**
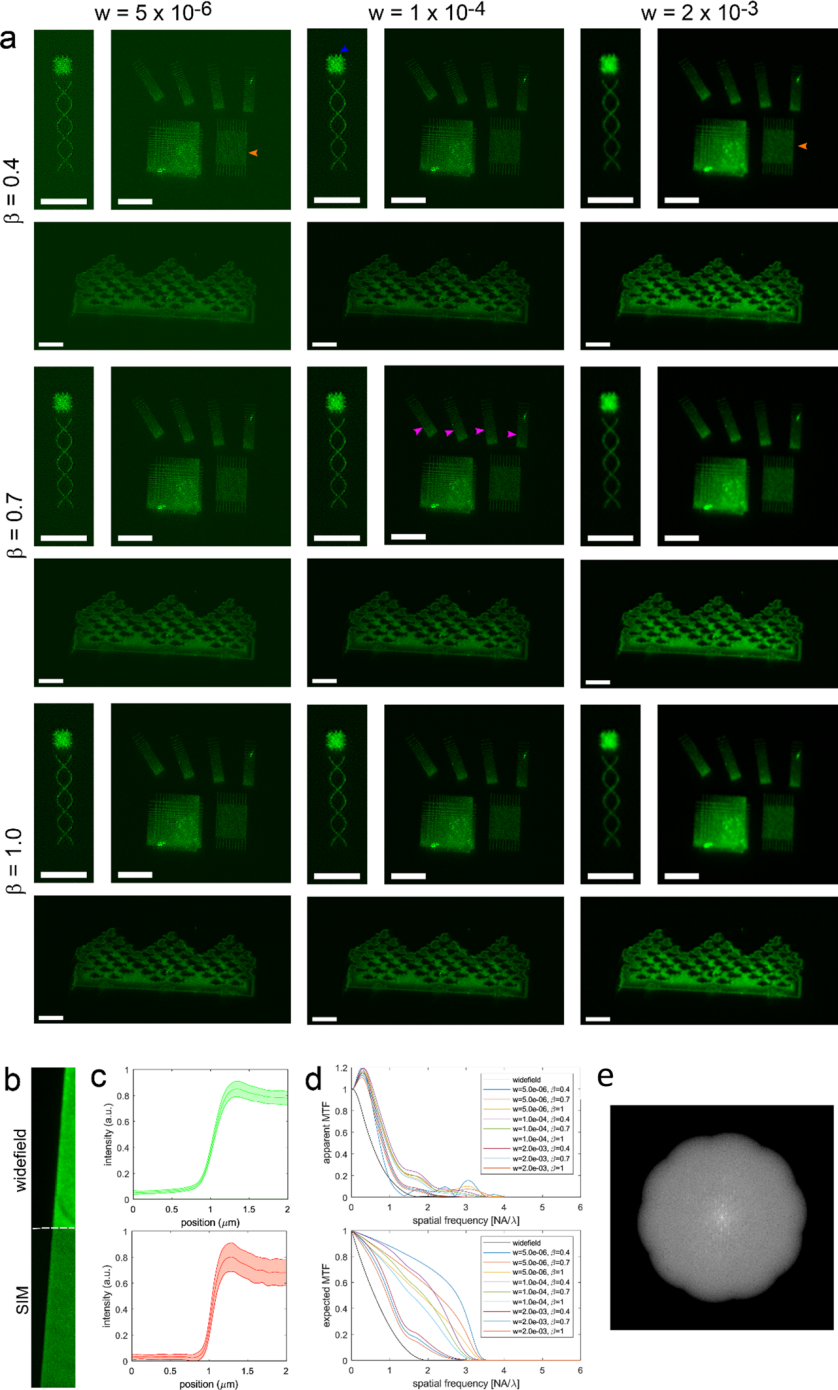
Assessment of SIM processing parameters. (**a**) 2D-SIM reconstructions of three nano-patterned structures for different regularization parameters $\mathrm{w}$ and for different exponents $\upbeta$ of the triangle apodization function. The fine grid (orange arrow) is resolved in SIM with low $\mathrm{w}$, but not with (too) high $\mathrm{w}$. The resolving power of SIM is isotropic, as can be seen from the just resolvable line spacing in the chirped grids with different orientations (pink arrows). For low $\mathrm{w}$ and for low $\upbeta$ contrast is higher, but at the expense of reconstruction induced noise. For balanced conditions the small lines on the sides of the small square (blue arrow) can be clearly recognized, for too low $\mathrm{w}$ the lines cannot be clearly separated from the noise structure, for too high $\mathrm{w}$ the small lines cannot be seen as was the case for wide-field (cf. Fig. 3a). The reconstruction induced noise is also visible in the background for low $\mathrm{w}$ and low $\upbeta$, but not for high $\upbeta$. (**b**) Imaged edge and (**c**) edge response (average and standard deviation indicated) for wide field and SIM ($\mathrm{w}=1\times {10}^{-4}$, $\upbeta =0.7$). (**d**) Apparent MTF derived from the edge response in (**c**), and expected MTF from reconstruction. The contrast and spatial frequency cut-off for SIM is improved compared to wide field. The reconstruction induced noise on the imaged plateau for low $\mathrm{w}$ makes the estimation of the MTF unreliable for this case. The overshoot at low spatial frequencies for SIM is probably due to a non-ideal underlying fluorescent edge object. A possible root cause is fluorescent material that accumulates close to edge. (**e**) Power spectral density on logarithmic scale for the Sky and Water I with $\mathrm{w}=1\times {10}^{-4}$, $\upbeta =0.7$. The periodicity in the pattern clearly appears as peaks in the power spectral density. Comparative images for the other parameter settings can be found in the Supplemental Figure S3. Scale bars in (**a**) are 3 µm, in (**b**) 2 µm.

SIM images obtained on our calibration samples were subjected to a set of standard reconstruction steps based on the generalized Wiener filter method^18^. This included setting a regularization parameter *w* and selecting a low-pass apodization filter for avoiding halo and edge ringing artefacts. The apodization was carried out using a triangle function characterized by an exponent $\beta$. Both *w* and $\beta$ parameters are selected by the user, for instance upon evaluation of the resulting reconstructed images, or pre-set in a generally applied reconstruction algorithm. Both have a clear impact on the quality of the resulting images, which is unambiguously visualized by conducting the SIM reconstructions for varying *w* and $\beta$ on the images of our calibration structures (Fig. 5a). For all images recorded with a relatively high value for *w*, a loss of high-resolution structural detail is clearly observed. This is particularly evident in the fine grid patterns. Setting the relatively lower value enhances noise in the final image. Similarly, for low $\beta$, reconstructed images show higher contrast at the expense of noise enhancement, which is particularly evident for both low $\beta$ and low *w*. Importantly, adjustment of both parameters may mask or enhance the appearance of features in the images as is most evidently illustrated in the reconstructions for the small protrusions in the small squares above the helices in Fig. 5a. For low *w*, the protrusions are hard to separate from the noise pattern within the squares, while for high *w*, they are blurred out like in wide-field microscopy (see also Fig. 3a). For balanced conditions, the protrusions can be clearly recognized. Without a priori known ground truth structure, parameter setting may thus lead to artefacts and can be prone to bias. Thus, our calibration structures clearly allow selecting optimized conditions ($w=1\times {10}^{-4}$, $\beta =0.7)$, balancing resolution loss and noise enhancement in the reconstruction. We note that these optimized settings may in general depend on signal to noise ratio and sparsity in the image and that the regularization may be automatically adjusted based on the image signal to noise ratio^19^.

The edge response at the large square marker used to identify the patterned areas in the fluorescence microscope (Fig. 5c) for both WF and SIM, enables extraction of the apparent MTF in both cases and, for SIM, also for the various combinations of *w* and $\beta$ (Fig. 5d). For SIM, the MTF is clearly extending to higher spatial frequencies. Also the artefacts for non-optimal values of *w* and $\beta$ are apparent in the MTF curves. We note that all SIM reconstructions display a similar overshoot in the low frequency regime, which may be due to a higher density of fluorescent molecules close to the edge. This could for instance occur as a result of proximity exposure. On writing a dense structure like the marker, proximity will effectively increase the dose deposited at each pixel. In conducting a dose series on an array of squares, we observed an optimal dose for functionalization beyond which the affinity for antibody labelling decreases and is ultimately inhibited (Supplemental Fig. S1). Thus, as our procedure was optimized for writing ~ 80 nm wide lines and small structures, proximity may cause the edge of a dense structure to exhibit higher binding affinity than the centre. The brighter edge of the Sky and Water I pattern could also be a result of this (Fig. 3c,f). This means that a further extension to more complex shapes or to even smaller feature size should include a calibration or estimation of proximity exposure in order to correct on a pixel-by pixel basis based on the aimed-for pattern dimensions similar to procedures in electron beam lithography.

## Conclusions

In conclusion, we have presented an optimized protocol for creating fluorescent nanostructures that can be used to evaluate and monitor the performance of super-resolution fluorescence microscopes and/or reconstruction algorithms. The ability to create complex two-dimensional nanostructures may help resolving feature versus artefact discussions that are hard to address using biological samples for which the precise nanoscale layout is not a priori known. As in our case electron microscopy images recorded after patterning reveal the dimensions of the areas to which the fluorescently labelled antibodies will bind, such a ground truth does exist. As a wide variety of fluorescent molecules can be used in combination with the standard immuno-labelling approach, we foresee the extension to multi-color samples and to the use of dyes used for single-molecule localization schemes, replacing the common practice of using simulated data sets for algorithm testing.

## Materials and methods

### Reagents

3-(Methoxy(polyethyleneoxy)propyl)trimethoxysilane, or PEG silane, 90%, 6–9 PE-units (CAS 65994-07-2) was obtained from ABCR GmbH, Germany. Anhydrous toluene, 99.8% (CAS 108-88-3) and triethylamine, 99.5% (CAS 121-44-8) from Sigma Aldrich Co., The Netherlands. A 100 nM solution of 2 g/L Rabbit anti-Mouse IgG (H + L) Cross-Adsorbed Secondary Antibody tagged with Alexa Fluor 488 (or IgG-Alexa488) was purchased from Life Technologies BV, The Netherlands. F(ab’)2-Rabbit anti-Goat IgG (H + L) Secondary Antibody Qdot 655 was also purchased from Life Technologies BV, The Netherlands. Indium-tinoxide coated glass slides (17 nm coating thickness as stated by supplier), 22 × 22 × 0.17 mm (OBL P/N 208965) were obtained from Optics Balzers, Switzerland.

### Patterning procedure

Below procedure has been previously documented in the first author’s PhD thesis^20^.

1. ITO-glass slides were cleaned by sonicating in acetone, followed by ethanol for 20 min each. They were then rinsed in deionised water and dried by wiping with lint free tissues.
2. 200 mL of a 20 nM solution of PEG silane was prepared by dissolving 2.36 g of PEG silane in 190 mL of toluene with 5% (10 mL) triethylamine as a catalyst.
3. The ITO-glass slides were exposed to an oxgen plasma at 200 W for 15 min in order to activate the surface.
4. Immediately after the plasma cleaning, the slides were immersed in the PEG-silane solution for 18 h at 60 °C to form a uniform PEG-silane monolayer on the ITO surface.
5. This was followed by a washing step to remove unbound PEG-silane. The slides were sonicated in toluene and then in acetone for 20 min each, rinsed in deionised water and dried as before.
6. The slides were then stored in dust-free boxes sealed with parafilm, taking care to ensure that the PEG layer did not make contact with any surface, including the box itself.
7. Next, slides to be patterned were mounted in the SEM and the pattering procedure was begun when the chamber pressure had reached approximately 1^.^10^–5^ mbar. Patterning was carried out at 5 keV and 25 pA. Care was taken to minimise vibrations (typically < 5 nm) and a fast beam blanker was used to prevent spurious patterning while translating the beam.
8. After noting the edge positions of the slide in the SEM, the slide was navigated to its centre position, where features where sought that could be used for focusing the beam. On a clean and uniform PEG silane layer typically such features are scarce, but a few small dust particles can mostly be located and serve as focusing markers.
9. Patterning was carried out using a home built Labview script which positioned the beam onto coordinates defined by the user in a script file. Patterning was always carried out blindly, i.e., care was taken not to expose on a field of view that was inspected prior to patterning. This was done to precisely control the electron dose for patterning as pre-inspection would result in an additional electron dose.
10. Large markers (about 200 µm) in size were first patterned in HR mode, next to which the arrays of high resolution patterns were made. The large markers serve for navigation in the fluorescence microscope or in the SEM for post-inspection. In order to maximize success and avoid, e.g., the presence of dust particles or surface non-uniformities on the patterned area, arrays always consisted of several repetitions of each pattern. Supplemental Fig. S2 shows two examples of pattern blocks with markers as used in this study.
11. After patterning, the sample was removed from the SEM and tagging of the patterns with an antibody bound to a fluorophore was performed. Hereto, a 100 nM solution was prepared by dissolving 375 µL IgG-Alexa488 in 50 mL of 1× TE buffer and pipetted out onto the sample drop by drop to cover the surface entirely. The sample was left to incubate for 45 min, in which time it was shielded from ambient light by covering with aluminium foil. For the quantum dots samples, a 1% solution of IgG-quantum dot in TE buffer was prepared by dissolving 20 µL in 200 µL of 1× TE buffer.
12. Finally, the slide was washed in TE buffer and rinsed with deionised water to remove non-specifically bound antibody molecules. It was dried following the same procedure described above, stored in a light-tight box to prevent photobleaching.

### Scanning electron microscopy

Patterning was performed in an FEI (now Thermo Fisher Scientific) Nova Nano 650 Dual Beam system, with conditions and settings as mentioned under step 6 in the patterning procedure above. Inspection was done using an FEI (now Thermo Fisher Scientific) Verios SEM at 5 keV beam energy and 100 pA current. Patterns were inspected in secondary electron mode using the in-lens (TLD) detector.

### SE and quantum dot line profile measurements

The SE line profiles were calculated by first rotating the SE images such that the lines are aligned in the vertical direction. Next, the images were summed across the vertical directions after which the following Gaussian function was fitted to each line:

$$f\left(x\right)=A*\mathrm{exp}\left(-\frac{{\left(x-{x}_{0}\right)}^{2}}{2{\sigma }^{2}}\right)+\mu$$

Individual quantum dots were filtered from the images using FiJi (ImageJ). First a variance filter of 2.0 px radius was applied, followed by thresholding such that in the 8-bit images pixels with value < 57 are discarded. Next, the binary image was eroded after which the Ultimate Points command was performed to reduce each detected quantum dot to a single point. A histogram of the x-values of these points in the image was obtained using Python, where the bin-size was set to approximately 10 nm.

### Fluorescence and structured illumination microscopy

RAW structured illumination images were acquired on a Zeiss Elyra PS.1 fitted with an Andor iXon 885 EMCCD camera with 1002 by 1004 pixels and a 63× Plan Apochromat lens with an NA of 1.4. The sample was illuminated with a High Power Diode Laser with a wavelength of 488 nm and a 495–575 nm bandpass emission filter. A grating was present in the light path and rotated 5 times and shifted 5 times resulting in 25 images per focal plane. A z-stack of 9 slices with 110 nm interval between slices was recorded and the focal slice with optimum pattern modulation contrast was selected for further SIM processing Settings were chosen to give the best images: EMCCD gain was kept low and saturation and bleaching were avoided.

Widefield images were recorded in widefield mode without the grating present in the light path.

Confocal images were acquired of the same sample with a Zeiss LSM780 confocal scanner fitted on the same microscope, using a 488 nm line of an argon laser. Signal was detected on a GaAsP detector with emission filtered between 500 and 550 nm. The pixel size for the confocal was matched to the reconstructed SIM image.

### SIM processing

The SIM reconstructions are made by a sequence of standard steps, according to the generalized Wiener filtering method^18^, implemented in the Zeiss Elyra code (Figs. 3 and 4), and with a home written code (Fig. 5). First, the raw images of a single focal slice of the data acquisition (5 orientations, 5 phases, so 25 raw images) are upsampled with a factor two using zero padding in the Fourier domain. Next, the peaks in the autocorrelation of low-pass filtered images are detected for finding the pitch and orientation of the illumination patterns. The phase of the autocorrelation peaks is used to estimate the phases of the illumination patterns^3,18,21–23^. The pattern phases are used to find the phase matrix, the pseudo-inverse of the phase matrix is used to transform the Fourier transforms of the raw images into the different image Fourier orders ${\widehat{I}}_{rm}\left(\overrightarrow{q}\right)$, ($r=\mathrm{0,1},\ldots ,5$ denotes the pattern orientation, $m=0,\pm\, 1,\pm\, 2$ the Fourier order, and $\overrightarrow{q}$ the spatial frequency vector, i.e. the position in Fourier space). The illumination pattern Fourier coefficients ${a}_{m}$ are found from the necessary consistency between the different image Fourier orders in the regions where they overlap. The image Fourier orders are low pass filtered with the (conjugate of the) microscope Optical Transfer Function (OTF) $\widehat{g}\left(\overrightarrow{q}\right)$, computed using a vectorial lens diffraction model as used by Righolt et al.^3^, and subsequently shifted to the correct position $m{\overrightarrow{k}}_{r}$ in Fourier space using the found values for pitch and orientation of the illumination pattern. Then, a weighted sum of the (low-pass filtered and shifted) image Fourier orders is taken, and a final Wiener filter is applied:

$${\widehat{I}}_{SIM}\left(\overrightarrow{q}\right)=\frac{\widehat{A}\left(\overrightarrow{q}\right)}{\widehat{D}\left(\overrightarrow{q}\right)+w}\sum_{r,m}{a}_{m}{\widehat{g}\left(\overrightarrow{q}+m{\overrightarrow{k}}_{r}\right)}^{*}{\widehat{I}}_{rm}\left(\overrightarrow{q}+m{\overrightarrow{k}}_{r}\right)$$

Here,

$$\widehat{D}\left(\overrightarrow{q}\right)=\sum_{r,m}{{a}_{m}}^{2}{\left|\widehat{g}\left(\overrightarrow{q}+m{\overrightarrow{k}}_{r}\right)\right|}^{2}$$

and $w$ is the so-called regularization parameter, needed to prevent division by zero for large values of of $\overrightarrow{q}$, and $\widehat{A}\left(\overrightarrow{q}\right)$ is the so-called apodization function, needed to prevent negative pixel values and edge-ringing artefacts^3,22^. We use an apodization function:

$$\widehat{A}\left(\overrightarrow{q}\right)={\widehat{\Lambda }\left(\overrightarrow{q}\right)}^{\beta }$$

where $\widehat{\Lambda }\left(\overrightarrow{q}\right)$ is the triangle function, which interpolates linearly between a value equal to one at the origin $\overrightarrow{q}=0$, and a value equal to zero at the SIM cutoff spatial frequency ${q}_{c}\left(\varphi \right)$ in the direction of $\overrightarrow{q}$, characterized by the azimuthal angle $\varphi$. Finally, an inverse Fourier transform of ${\widehat{I}}_{SIM}\left(\overrightarrow{q}\right)$ results in the SIM reconstruction ${I}_{SIM}\left(\overrightarrow{q}\right)$. A wide field equivalent image can be found by simply adding all raw images.

The MTF can be measured from sharp edges that are imaged. Briefly, image lines are upsampled (with a factor 4), shifted to have the edges overlap, added to reduce noise, differentiated to get the edge response function, and Fourier transformed.

For the power spectral density, we plotted log(1 +|FT(image)|^2^) where FT stands for the Fourier transform.

The displayed figures in Fig. 5 show the full dynamic range of the images, with the exception of the nano-patterned structure with the patterning defect, which is clipped to make the actual structure visible.

## Supplementary Information

Supplementary Information.

## Data Availability

Datasets generated during this study are available from the corresponding author upon reasonable request.
