# Supplementary material for: Electron-beam patterned calibration structures for structured illumination microscopy
Source: Sci Rep. 2022 Nov 23;12:20185. doi: 10.1038/s41598-022-24502-0 (PMC9684522; doi:10.1038/s41598-022-24502-0)
Supplement: Supplementary file 1 — Supplementary Information. [file 41598_2022_24502_MOESM1_ESM.docx]

**Supplemental Information**

**Electron-beam patterned calibration structures for structured illumination microscopy.**

Sangeetha Hari^1^, Johan A. Slotman^2^, Yoram Vos^1^, Christian Floris^1^, Wiggert A. van Cappellen^2^, Kees (C.) W. Hagen^1^, Sjoerd Stallinga^1^, Adriaan B. Houtsmuller^2^, and Jacob P. Hoogenboom^1^

^1^ Imaging Physics, Delft University of Technology, Delft, the Netherlands

^2^ Department of Pathology, Erasmus Optical Imaging Centre, Erasmus Medical Center, Rotterdam, The Netherlands.

**Influence of substrate quality on patterning results page 1**

**Electron-dose influence on patterning and functionalization mechanism page 2**

**Example lay-out of patterning block page 6**

**Power spectral density for different regularization parameters page 7**

**Influence of substrate quality**

Choice and quality of the underlying substrate for grafting the PEG-silane self-assembled monolayer (SAM) was found of critical importance to ensure reproducible patterning results, in line with an earlier study investigating growth of an octadecylphosphonic acid SAM on ITO [1]. We conducted test experiments with two different commercially available ITO substrates, and a third variant where we conducted an additional silicon oxide coating. The as-received commercial substrates were as follows:

ITO-1: Glass slides with a 17 nm thick ITO layer (SPI supplies #06494-AB: ITO coated cover slips, size: 22X40 mm, thickness #1: 0.13-0.17 mm, resistivity: 70-100 ohms/square, nominal transmittance:

88%, all data as stated by the supplier)

ITO-2: Glass slides with a 17 nm thick ITO layer (Optics Balzers cover glass 22X22X0.17 mm with ITO, OBL P/N 208965). These slides were previously used and characterized by our group [2].

ITO-3: ITO-2 coated with ˜10 nm thick silicon dioxide layer deposited by sputtering.

ITO-3 was added in this manner because based on the results of [1] the amorphous SiO_2_ resulting from sputter deposition would be expected to lead to better PEG quality. We found the thickness of 10 nm to be thin enough to prevent charging of the sample. Patterning on ITO-2 and ITO-3 was found to yield highly reproducible results both over a large area of the sample as well as over different samples. ITO-2 was therefore selected for all subsequent experiments and ITO-3, while found satisfactory and potentially beneficial to avoid fluorescence quenching by the ITO [2], was not used due to the additional manufacturing step.

**Electron-dose influence on patterning and functionalization mechanism.**

A detailed characterization of the dimensions of the fluorescence nanopatterns as a function of electron dose was carried out. We patterned series of lines, squares, dots with 5 keV and 25 pA electron beam where the dose was varied from 50 C/m^2^ to 500 C/m^2^ in steps of 50 C/m^2^ by increasing the number of passes, using a fixed dwell time of 52.4 µs. All samples were tagged with IgG-Alexa 488 after patterning following the above procedure.

Fig. S1 shows an image of two arrays of squares after electron beam patterning with increasing electron dose and subsequent fluorescence functionalization. The image has been acquired on a Nikon Eclipse fluorescence microscope with a 100X oil immersion objective. For low doses (50 C/m^2^, 100 C/m^2^), we observe homogeneous fluorescence over the entire patterned square, becoming more intense with dose. However, we then observe a turning point at 150 C/m^2^, where the intensity in the centre of the square starts to decrease, and the edges begin to appear fluorescent. This is verified by taking integrated line scans for each row along the length of the yellow rectangles (Fig. S1(b) and (c), for Row 1 and Row 2 respectively).

We attribute the observation of an optimum dose for functionalization, followed by inhibition of functionalization to different regimes in the electron beam induced modification of the PEG layer, followed by complete dissociation and possibly deposition of decomposed other hydrocarbons present in the SEM vacuum chamber. Electron-beam induced PEG modification has been previously studied in detail by Rundqvist et al.[3]. They observed that PEG undergoes different modifications depending on the total electron dose used. Moreover, Schlapak et al. observed the functionalization on electron-beam exposed PEG to be a charge-mediated interaction resulting from induced negative surface charge with positive charged groups on immobilized bio-molecules [4]. However, in their case the negative surface charge was attributed to electron-beam induced deposition of hydrocarbons present in the vacuum chamber onto the PEG SAM, while we find direct PEG modification followed by dissociation and deposition to better explain our observation of a dose-dependent optimum. Thus, we can discern 3 different regimes in electron dose, characterized by different stages of electron-beam induced modification as follows:

Regime 1: Low dose (<120 5keV electrons/nm^2^)

Primary electrons (PEs) and low energy secondary electrons generated by the PEs chemically modify PEG in the exposed area. The total dose is such that some PEG molecules acquire a probably negative net charge in the buffer solution used for antibody functionalization, making the exposed area suitable for antibody binding. This results in the fluorescence uniformly filling the exposed square.

Regime 2: Intermediate dose (120 to 600 5keV electrons/nm^2^)

Increasing dose increases the amount of modified PEG molecules in the exposed area, increasing the probability for antibody attachment. This results in increasing fluorescence intensity with increasing dose.

Regime 3: High dose (> 600 5keV electrons/nm^2^)

Continued electron exposure leads to multiple chemical reactions taking place in the PEG monolayer and/or absorbed hydrocarbons, leading to dissociation and carbon deposition. Antibody binding on the PEG is increasingly hampered by hydrophobic carbon patches. Also, fluorescence quenching by carbon nanodeposits might not be excluded as a contributing factor. Together, this leads to decreasing fluorescence intensity with increasing electron dose. At the same time, the region surrounding the patterned area has not been exposed to the PEs directly, but undergoes an increasing proximity exposure to SEs generated during patterning. This region is therefore modified suitably for antibody binding and fluorescence from the edges is observed, going up with dose as in Regime 2.

We note that in the SEM images of low-dose exposed areas in Fig. 1b in the main manuscript, contrast speckle is visible, which may be attributed to shot noise in the exposure in line with the regime 1 and regime 2 mechanism. Further, in the quantum dot functionalized areas in Fig. 2, quantum dots appear bound on the slightly darker patches, although we cannot exclude that here substrate contrast is influenced by the presence of the quantum dot. An additional experiment carried out with gold nanoparticle functionalization followed by SEM analysis (Fig. S2) shows the same proximity trend as that observed for fluorescence in Fig S1 as well as similar binding to patches that appear darker in SE contrast as observed for quantum dots in Fig. 2.

We further note that the trends observed on the square patterns in Fig S1 are not visible in the line patterns because their width is too low to permit these details to be resolved with fluorescence microscopy. However, we expect the above regimes and their physical mechanisms to still apply and thus that line profiles undergo the same trends, with the transitions occurring at a different dose because the patterning strategies for lines and squares differ. An important conclusion of this study is that the choice of dose can be significant in obtaining patterns of not only the desired size, but also the desired intensity distribution over the pattern. For the fabrication of high resolution, uniformly fluorescent structures, low doses (< 100 C/m^2^) must be used. Moreover, further extension of this technique to smaller structures and/or smaller line spacing may benefit from detailed dose deposition calculations including proximity effects.

a

**
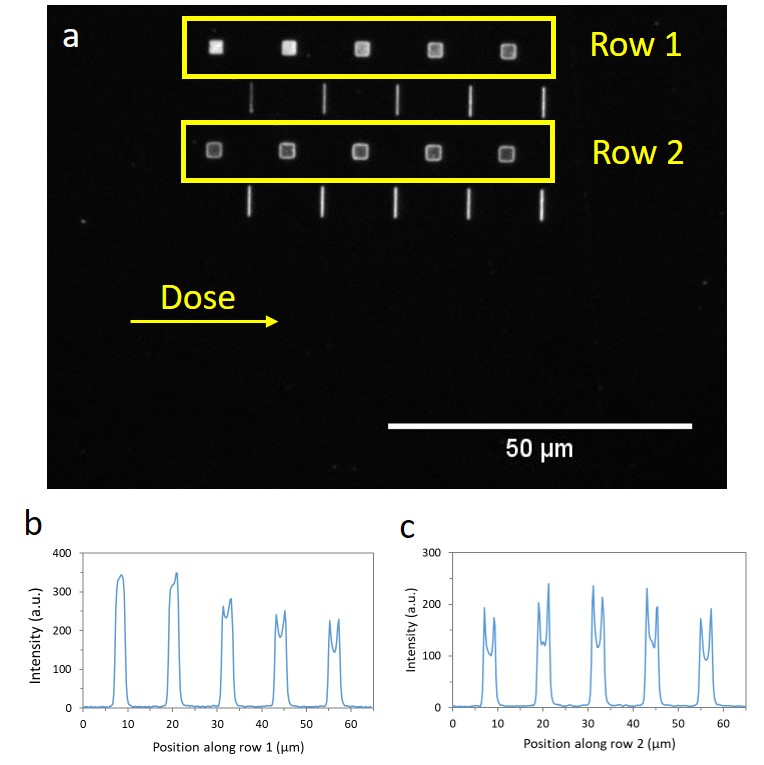
**

**Figure S1 Increasing electron dose first enables then inhibits functionalization.** *(a)* *Two rows of square and line patterns exposed with, from left to right and from row 1 to row 2, increasing electron dose (Row 1: 50 C/m^2^ to 250 C/m^2^; Row 2: doses 300 C/m^2^ to 500 C/m^2^, with 50 C/m^2^increments (left to right). (b) Fluorescence intensity integrated along the patterns marked as Row 1, and (c) as Row 2. From the images and intensity profiles it is apparent that low dose allows for fluorescence functionalization, while increasing dose leads to inhibition, first in the center and then gradually moving outward as a result of proximity exposure.*


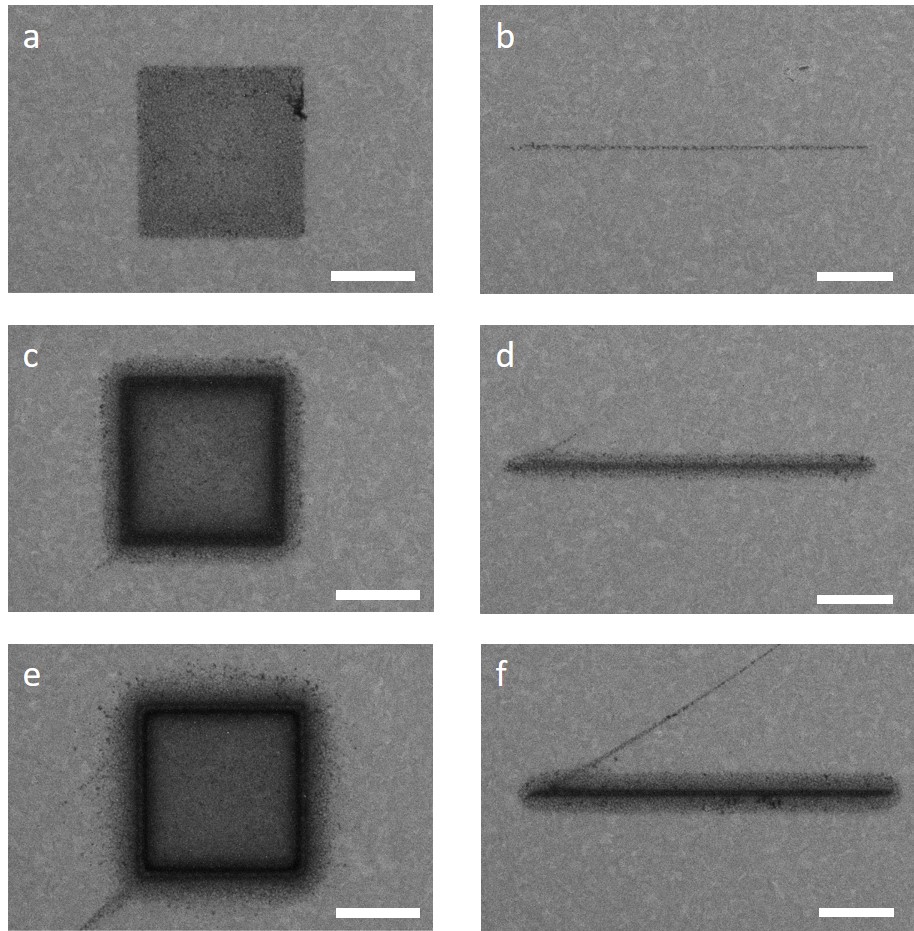


**Figure S2 Gold nanoparticle functionalization shows similar trends as fluorescent dye and quantum dot functionalization.** *Gold nanoparticle labelling on patterns of squares and lines exposed with electron dose of (a, b) ~10 C/m^2^, (c, d) ~200 C/m^2^, (e, f) ~1000 C/m^2^. Functionalization is clearly restricted to the areas that appear darker in secondary electron contrast. With increasing dose, both inhibition of functionalization and proximity effects are observed. The streak lines that can be observed in the higher dose exposed areas at the lower left corner of the squares (c, e) and the left corner of the lines (d, f) result from beam translation between patterns. Scalebars 1 µm.*

**Figure S3. Example layout of patterning blocks with markers and dimensions as used in this study.** *(a) Several repetitions of the array of alternating long and short lines with decreasing spacing (comprising Block A) and the two-dimensional checkerboard array (comprising Block B) alongside a large homogeneous marker (Marker 1). (b) Several repetitions of arrays of lines at decreasing spacing under different rotation angles (comprising Block C), alongside a differentiating marker (Marker 2). Final patterning scales as indicated in the figures.*

**
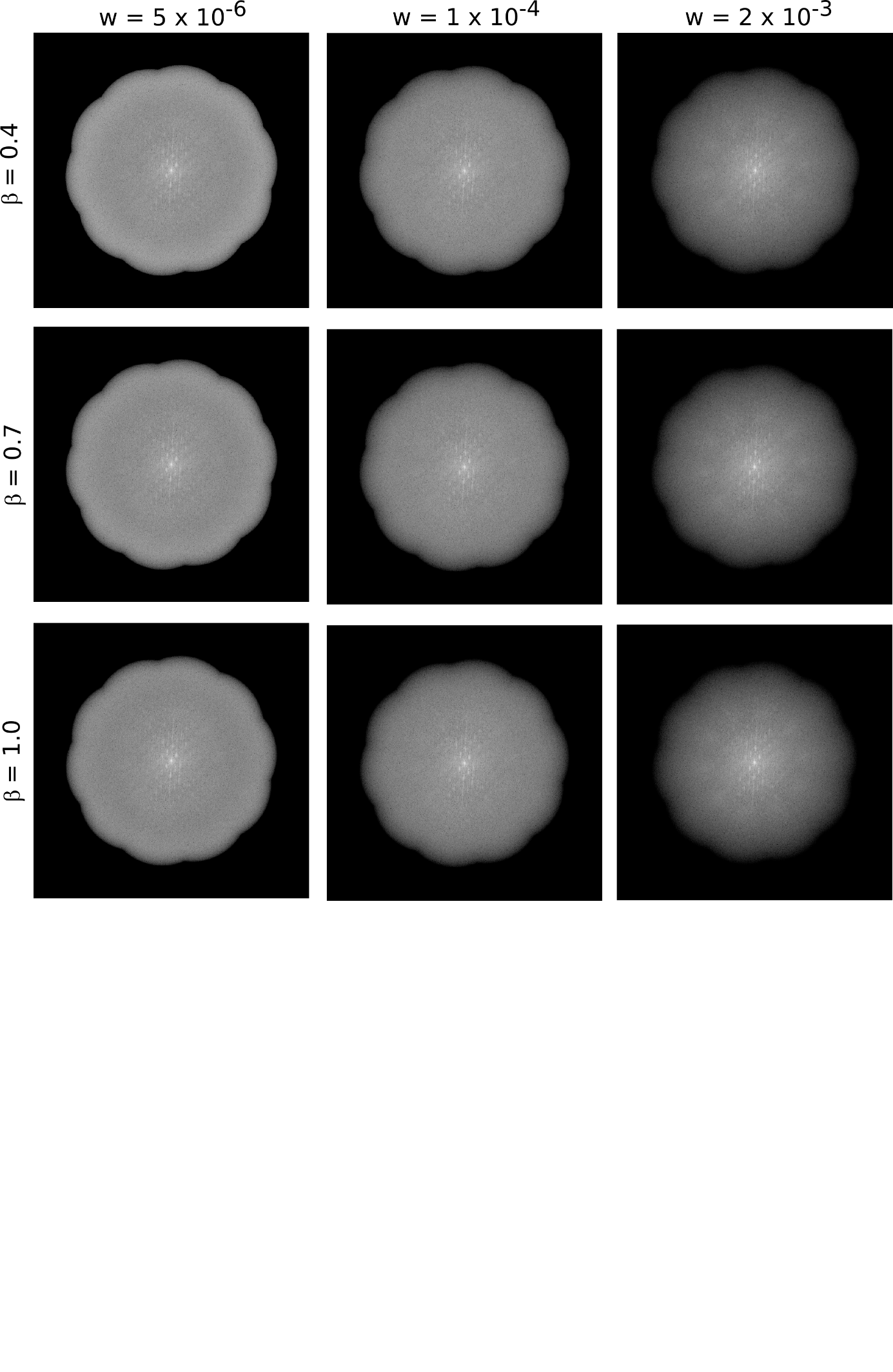
**

**Figure S4. Power spectral density for the Sky and Water I patterns reconstructed with varying values for the regularization parameters *w* and *β.*** *Higher spatial frequencies appear for lower regularization parameters as expected.*

**References**

[1] M. Chockalingam, N. Darwish, G. Le Saux, and J. J. Gooding, “Importance of the Indium Tin Oxide Substrate on the Quality of Self-Assembled Monolayers Formed from Organophosphonic Acids,” *Langmuir*, vol. 27, pp. 2545–2552, 2011.

[2] R. J. Moerland and J. P. Hoogenboom, “Subnanometer-accuracy optical distance ruler based on fluorescence quenching by transparent conductors,” *Optica*, vol. 3, no. 2, 2016.

[3] J. Rundqvist, J. H. Hoh, and D. B. Haviland, “Directed Immobilization of Protein-Coated Nanospheres to Nanometer-Scale Patterns Fabricated by Electron Beam Lithography of Poly( ethylene glycol) Self-Assembled Monolayers,” *Langmuir*, vol. 22, no. 11, pp. 5100–5107, 2006.

[4] R. Schlapak, J. Danzberger, T. Haselgrübler, P. Hinterdorfer, F. Schäffler, and S. Howorka, “Painting with biomolecules at the nanoscale: Biofunctionalization with tunable surface densities,” *Nano Lett.*, vol. 12, no. 4, pp. 1983–1989, 2012.
